# SGLT2 inhibitor therapy and pulmonary artery pressure in patients with chronic heart failure—further evidence for improved hemodynamics by continuous pressure monitoring

**DOI:** 10.1007/s00392-021-01954-4

**Published:** 2021-10-24

**Authors:** Klara Kirschbaum, Mariuca Vasa-Nicotera, Andreas Michael Zeiher, Sebastian Cremer

**Affiliations:** 1 Department of Medicine III, Cardiology/Angiology/Nephrology, Goethe University of Frankfurt, Theodor-Stern-Kai 7, 60590 Frankfurt, Germany; 2 German Center for Cardiovascular Research DZHK, Berlin, Germany; 3 Partner Site Rhine-Main, Mainz, Germany; 4 Cardiopulmonary Institute, Goethe University Frankfurt, Frankfurt, Germany

**Sirs:**

SGLT2 inhibitors were designed as drugs for patients with diabetes that interfere with renal glucose and sodium reabsorption. The EMPEROR-reduced and DAPA-HF trials showed that SGLT2i treatment led to a significant reduction in the combined endpoint of cardiovascular death and hospitalizations in patients with heart failure with a reduced ejection fraction independent of diabetes status [1, 2]. However, the mechanisms responsible for these effects remain largely unexplored and clinical observations assessing heart function or hemodynamic status of those patients remain scarce.

A key feature of heart failure is inadequate cardiac output resulting in congestion and secondary pulmonary hypertension [3]. Higher pulmonary artery pressures predict HF symptoms, unplanned hospitalizations and mortality [4, 5]. Therefore, we analyzed the effect of SGLT2i on PA pressures in patients with severe heart failure regardless of ejection fraction. For this purpose, we analyzed data recorded by the CardioMEMS system, a remote sensor which transmits ambulatory recorded pulmonary artery pressures.

This was a retrospective case-series of ambulatory HFrEF or HFpEF patients who had previously received a CardioMEMS device, in whom either Dapagliflozin or Empagliflozin was initiated at the discretion of the treating physician. Hemodynamic data were abstracted from the medical record. PAP readings were collected daily starting 4 weeks prior to SGLT2i initiation for up to 10 weeks following initiation of treatment with SGLT2i. Patient records were audited to verify that other interventions that might have influenced PAP measurements (e.g., addition of new diuretic drugs and change in diuretic dose) had not occurred during the observation period.

All patients were on stable guideline-directed medical therapy for at least three months prior to SGLT2i treatment initiation. Adjustment of standard therapies was permitted if clinically indicated. Patients were excluded if they had initiation of β-blockers, angiotensin-converting enzyme inhibitor/angiotensin II receptor blockers, or valsartan/sacubitril within three months before SGLT2i initiation.

Hemodynamic data pre and post SGL2 initiation were compared via Wilcoxon signed-rank testing and linear regression analysis was employed. All patients provided written informed consent for participation in this single center registry (NCT03020043). The study complies with the Declaration of Helsinki and was approved by the local ethics committee.

Statistical significance was assumed if *P* < 0.05. Statistical analysis was performed with Graph Pad Prism Vers. 8.2.0.

**Figure1:**
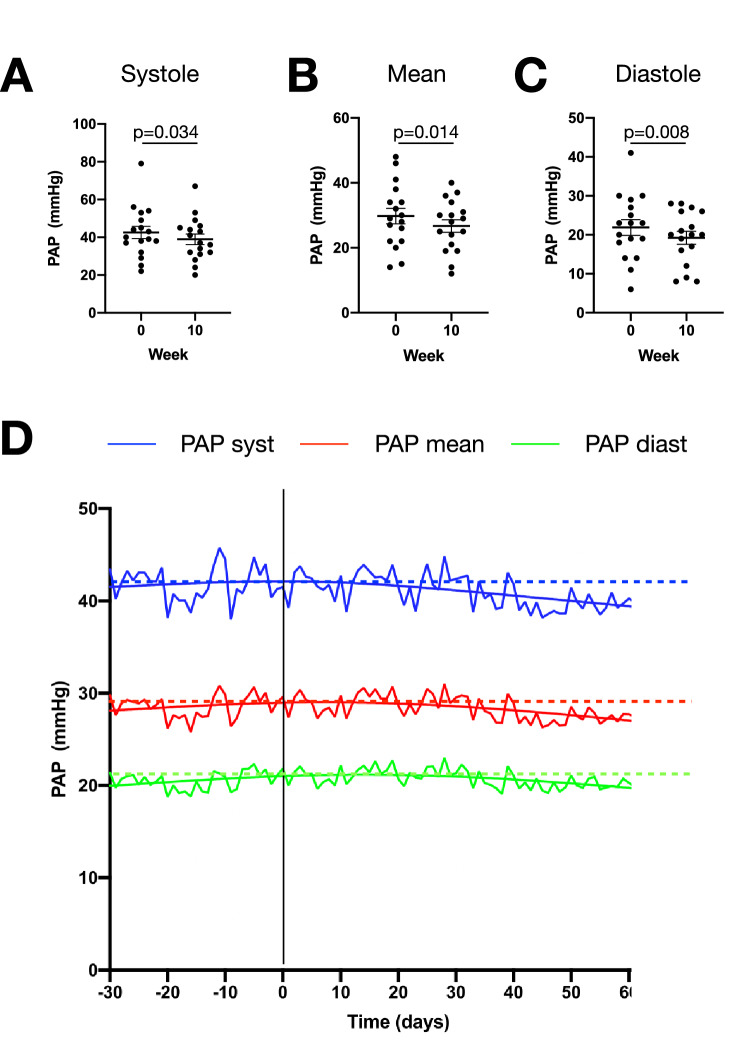
Systolic (**A**), mean (**B**) and diastolic (**C**) pulmonary artery pressures at baseline and 10 weeks after initiation of a SGLT2 inhibitor. *P* values are indicated. Wilcoxon signed rank test. **D** Pulmonary artery pressure evolution from 30 days prior to SGLT2i start until 70 days after initiation of a SGLT2 inhibitor. Shown are mean daily values of all patients for systolic, mean and diastolic pulmonary artery pressures with spline fitting curve. Vertical bar, timepoint of SGLT2i initiation; horizontal dashed bars, mean PAP values at timepoint of SGL2i initiation. *P* < 0.0001 in linear regression analysis from day 0 to day 70 for all PAP values

In the present cohort, 17 patients with advanced chronic heart failure were analyzed. All patients underwent implantation of a CardioMEMS system between 2015 and 2020. SGLT2 inhibitors were initiated either as diabetes therapy or as a heart failure drug. 8 of 17 patients had diabetes**.** 13 patients received dapagliflozin (1 × 10 mg/d), four patients were treated with empagliflozin (1 × 10 mg/d). Baseline characteristics and medication of all patients are shown in Table1. PAP values were recorded on a daily base in the 30-day period prior to initiation of SGL2i treatment until 70 days (10 weeks) after start of SGLT2 intake. After 10 weeks, we observed significant reductions of systolic PAP (− 3.59 ± 1.55 mmHg; *P* = 0.034), mean PAP (− 3.06 ± 1.22 mmHg; *P* = 0.014), and diastolic pulmonary artery pressure (− 2.65 ± 0.98 mmHg; *P* = 0.008; Fig. 1A–C). Off note, dosage of loop diuretics did not change significantly in the observation period (Table 1). Other heart failure medications were not relevantly changed in the 30-day period prior to SGLT2i start and in the 70 days after drug initiation. Interestingly, PAP changes occurred already after 3 weeks of treatment and increased over time (*P* < 0.001 for all PAP values, Fig. 1D).

**Table 1:** Baseline characteristics

Demographics		
Age (y)	67.18 ± 3.09	
Male, *n* (%)	17 (100)	
Medical history		
HFrEF, *n* (%)	15 (88.2)	
HFpEF, *n* (%)	2 (11.8)	
Ischemic heart disease, *n* (%)	15 (88.2)	
Diabetes mellitus, *n* (%)	8 (47.1)	
NYHA class	2.56 ± 0.12	
Baseline laboratory values		
nt-proBNP (pg/ml)	1916 ± 1050	
Creatinine (mg/dl)	1.42 ± 0.11	
Baseline Heart Failure Medication		
Betablocker, *n* (%)	17 (100)	
ARNI, *n* (%)	15 (88.2)	
ACE inhibitor, *n* (%)	2 (11.8)	
MRA, *n* (%)	13 (76.4)	
Torasemide, *n* (%)	17 (100)	
Torasemide dose		
30 days prior to SGLT2i start (mg)	45.29 ± 12.75	
Baseline (mg)	45.59 ± 10.2	
70 days after SGLT2i start (mg)	41.18 ± 12.24	*p* = 0.88

Values are shown as absolute numbers (percentages) and mean ± SEM

In this single-center, non-randomized observational study, the addition of a SGLT2i to optimal medical therapy consisting of an ARNI, betablocker and aldosterone-antagonist significantly decreased PA-pressures in patients with heart failure. This effect commenced after 3 weeks and increased over time, reaching 3 mmHg after 10 weeks. The results were consistent for PA diastolic, systolic and mean pressures. The presence of pulmonary hypertension, which occurs in 40–75% of patients with HFrEF and 35–80% of patients with HfpEF [3], is associated with higher frequency of heart failure hospitalizations and increased mortality [5]. Inclusion of PA-Pressures as an additional treatment target substantially reduced hospitalizations for heart failure in both HFpEF and HfrEF [6, 7].

Our results add to a smaller observation which describes lower PA pressures within 1 week after initiation of dapagliflozin in 9 patients assessed by remote PA-pressure sensors[8] and confirm data from a small, randomized study, in which 33 patients who started empagliflozin recorded lower PA pressures within a 12-week timespan [9].

The mechanisms contributing to the beneficial effect of SGLT2i in heart failure remain elusive. Possible mechanisms for the effects observed in our study suggest either direct effects on the heart, vasodilation of the pulmonary vasculature, a direct natriuretic/diuretic effect or a combination of those as possible causes for improved hemodynamics in patients with HF. Indeed, it was shown that empagliflozin might improve diastolic function of human myocardium, while systolic contractility was not affected [10]. Correspondingly, empagliflozin significantly reduced the PCWP after 12 weeks of treatment in patients with HFrEF in a small clinical trial [11]. Moreover, experimental studies describe attenuation of vascular dysfunction and enhanced endothelium-dependent vasorelaxation, mechanisms, which are relevant in pulmonary hypertension due to heart failure [12]. Finally, two randomized trials examining the effects of empagliflozin on cardiac remodeling in patients with HFrEF reported improvements of left ventricular volumes and function after 6 months of treatment [13, 14]. This might have been driven by either a direct effect of SGLT2i on the myocardium or an improvement of volume status. The effects observed in the present manuscript developed rather early, suggesting that a diuretic effect at least in part contributed to improved hemodynamics after initiation of an SGLT2i. Indeed, a small clinical study showed that empagliflozin causes natriuresis and reduces blood volume. This mechanism does not come with the cost of neurohumoral activation, reduction in kidney function or electrolyte wasting, which are common side effects of classical diuretics [15]. Off note, dosage of diuretics did not change significantly over the observation period in our study, which corroborates data from the DAPA-HF trial [16]. In addition, the reduction of PA-Pressures observed in our cohort occurred in patients with optimal medical background therapy. All patients were treated with betablockers and RAAS-blockade, and 76% of all patients were treated with an aldosterone receptor antagonist.

This study is a small, single center, non-randomized trial. However, it extends previous observations and frequent assessment of PA pressures allowed the generation of significant data. In summary, an early and persistent fall in PA pressures occurs after addition of an SGLT2i to optimal medical therapy in patients with heart failure, which, therefore, should be considered as a first line heart failure treatment.
